# Imiquimod-induced pruritus in female wild-type and knockin Wistar rats: underscoring behavioral scratching in a rat model for antipruritic treatments

**DOI:** 10.1186/s13104-023-06627-1

**Published:** 2023-11-25

**Authors:** Karen Lariosa-Willingham, Dmitri Leonoudakis, Florian Simon, Kendall Walker, Philippe Guillaume, Liling Warren, Jennifer Stratton

**Affiliations:** 1 Teva Pharmaceutical Industries Ltd, Redwood City, CA 94063 USA; 2 Porsolt SAS, ZA de Glatigné, 53940 Le Genest-Saint-Isle, France

**Keywords:** Psoriasis, Imiquimod, Pruritus, Wistar rat, Scratching bout

## Abstract

**Objectives:**

Animal models of skin disease are used to evaluate therapeutics to alleviate disease. One common clinical dermatological complaint is pruritus (itch), but there is a lack of standardization in the characterization of pre-clinical models and scratching behavior, a key itch endpoint, is often neglected. One such model is the widely used imiquimod (IMQ) mouse model of psoriasis. However, it lacks characterized behavioral attributes like scratching, nor has widely expanded to other species like rats.

Given these important attributes, this study was designed to broaden the characterization beyond the expected IMQ-induced psoriasis-like skin inflammatory skin changes and to validate the role of a potential therapeutic agent for pruritus in our genetic rat model. The study included female Wistar rats and genetically modified knockin (humanized proteinase-activated receptor 2 (F2RL1) female rats, with the widely used C57BL/6 J mice as a methodology control for typical IMQ dosing.

**Results:**

We demonstrate that the IMQ model can be reproduced in rats, including their genetically modified derivatives, and how scratching can be used as a key behavioral endpoint. We systemically delivered an anti-PAR2 antibody (P24E1102) which reversed scratching bouts—validating this behavioral methodology and have shown its feasibility and value in identifying effective antipruritic drugs.

**Supplementary Information:**

The online version contains supplementary material available at 10.1186/s13104-023-06627-1.

## Introduction

The IMQ murine model of acute skin inflammation has been widely used for preclinical studies of psoriasis since 2009. Briefly, daily topical doses for 5–6 days of 5% IMQ cream to shaved back skin of mice induces localized, systematic inflammation primarily through toll-like receptor (TLR) 7/8 activation resulting in erythema, scaling, keratinocyte proliferation and a dermal infiltrate that includes T lymphocytes [1]. Macroscopically this results in flaky patches, discolored scaly skin and intense itchiness or pruritus—characteristics consistent with human psoriasis lesions and other models of cutaneous lesion development [2, 3]. Furthermore, patients often experience unavoidable, uncontrollable itchiness and scratching aggravates existing skin lesions. Therefore, treatments that alleviate itching in this disease are essential. Finally, the reported existence of common pathways between psoriasis and other lesional skin diseases means that the IMQ is also used for other skin diseases [4].

However, frequently these studies overlook the pruritus-induced scratching, biting and licking behaviour [5] and due to the scarcity of behavioural endpoint data in rats they are rarely used in dermatology models. Here we developed a model of topically applied IMQ to rats, validating the induction of scratching behaviour, skin lesions, and further correlating dermal thickening with histology. Furthermore, we tested a genetically modified rat human F2RL1 knockin strain to demonstrate the efficacy of a topical corticosteroid and systemic delivery of the P24E1102 antibody antagonist to reduce IMQ-induced lesions and scratching. Our results aligned with a recent investigation in Wistar rat [6] in terms of duration of induction, surface area of psoriatic lesions, histopathology and pathways involved.

## Materials and methods

### Animals

F2RL1 KI Wistar female rats (Teva Pharmaceutical Industries Ltd.), Wistar rats and C57BL/6 J mice about 12 weeks old (Janvier Labs, France) were housed five days prior to experimentation (Porsolt Research Laboratory, Le Genest-Saint-Isle, France). All animals were handled according to the institutional guidelines (Declaration of Helsinki, Directive No. 2010/63/UE, Decree No. 2013–118; Association for Assessment and Accreditation of Laboratory Animal Care (AAALAC International; ARRIVE 2.0). The study procedures were approved by the Internal Animal Care and Use Committee (IACUC, Porsolt Code n°F53 1031). Animal housing was maintained in a light, humid and temperature-controlled environment (12 h, 40–70%, 22 ± 2 °C). All animals had access to food and water ad libitum. Environmental enrichment was provided. Euthanasia was performed by exposure to carbon dioxide following blood sampling.

## Materials

### Preparation of full-length antibodies and F(ab)’2 fragments

P24E1102, a fully humanized monoclonal antibody (IgG4, kappa) was used as an investigative article in our experiments. MOPC is an isotype-matched control. (Teva Pharmaceutical Industries Ltd., Patent Application WO2022040345A1) The F(ab)’2 fragment of P24E1102 was used as a tool to investigate the selective targeting of peripheral administered dosing without the possible immunomodulatory effects of the Fc region of the full-length antibody. Briefly P24E1102 and MOPC antibodies were digested with pepsin, subject to affinity chromatography purification resulting in F(ab)’2 fragments using sulfate columns. Purity and integrity of the F(ab)’2 was analysed by SDS-PAGE under reducing and non-reducing conditions. Functional activity was confirmed by attenuation of calcium influx in PAR2 expressing cell lines.

### Compound administration

**Fig. 1:**
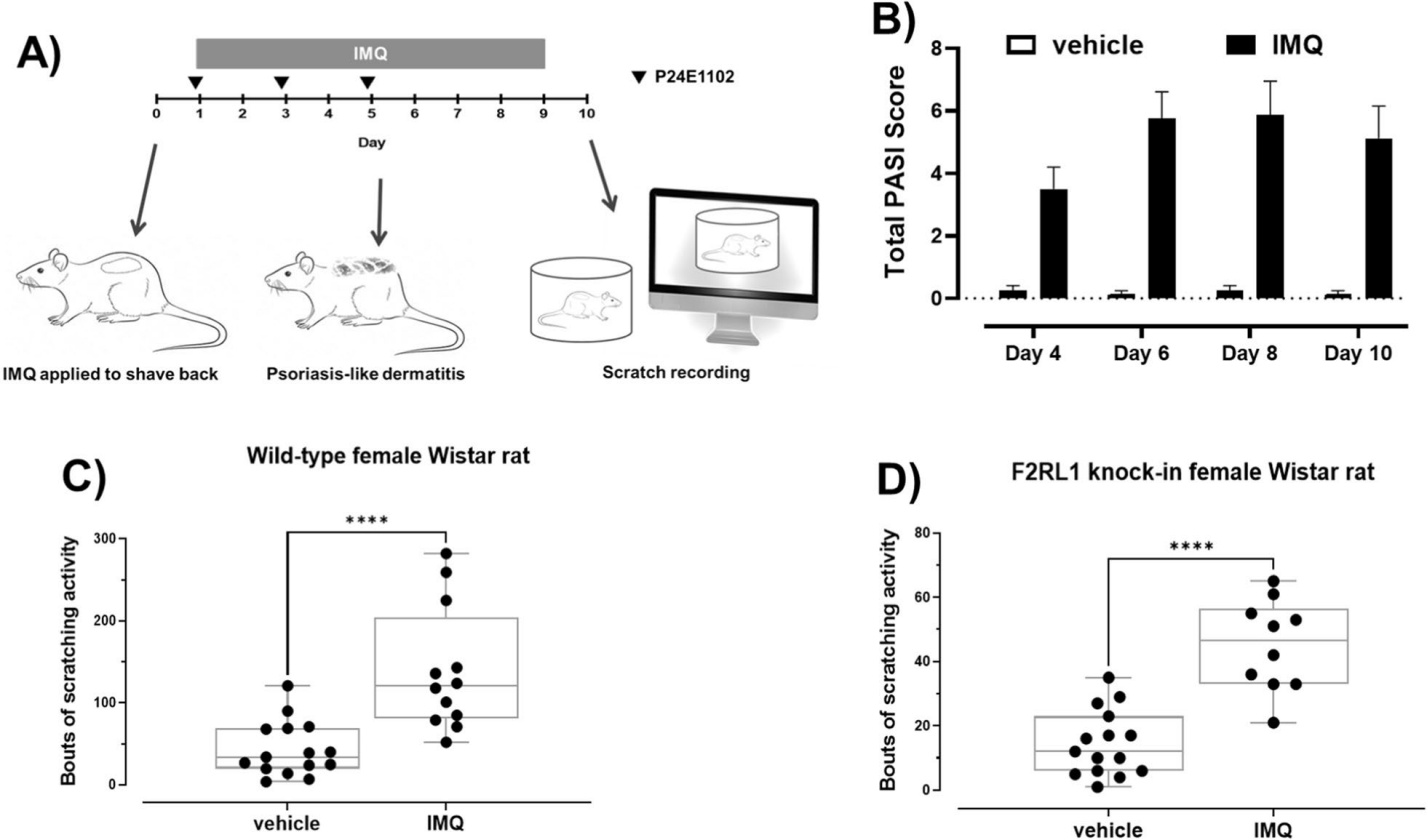
Study design. **A** Female Wistar rats received from nine daily topical administrations of imiquimod (IMQ) or vehicle (Vaseline^®^). Test antibodies or vehicle formulations were administered on Day 1, 3, and Day 6. Development of psoriasis-like dermatitis **B** appeared starting Day 4 continuing through Day 10. On Day 10, severity of the back inflammation was monitored on a modified clinical Psoriasis Area and Severity Index (PASI) scoring system. Scratching behavior was captured by videotape and serum and back skin samples were collected for cytokine measurements and immunohistochemistry to visualize IMQ induced morphological changes. Number of scratching bouts in IMQ-treated rats was significantly (P < 0.0001) higher than animals exposed to vehicle in **C** female Wistar rats and **D** F2RL1 knockin female Wistar rat animals from various studies under control conditions (n = 15 total). Data is expressed as the mean (SEM). Asterisk (*) denotes P values of IMQ compared to vehicle, *P < 0.0001 (Student’s *t*-test, followed by Mann–Whitney post-test)

Up to 30 animals (depending on the study) were randomly distributed to groups of 5–6 animals per treatment housed in separate cages. Single housed animals received daily topical doses of 37.5 mg/cm^2^ of 5% IMQ cream (Aldara^™^, 3 M Pharmaceuticals, St. Paul, MN) or control vehicle (Vaseline, Unilever, USA) on the shaved back for 9 consecutive days. This translates to total area coverage of about 150 mg of IMQ cream or vehicle onto an area of 4 cm^2^ dilapidated skin. Antibodies P24E1102 and MOPC (30 mg/kg) were administered (Fig. 1A) by intravenous route (IV) to animals one hour prior to each IMQ administration. F(ab)’2 fragments of P24E1102 and MOPC were administered similarly using a molar equivalent of 30 mg/kg. In one treatment group, only clobetasol (0.05% DERMOVAL), a standard-of-care corticosteroid for treating dermal swelling and itching, was topically applied (120 mg) one hour after every IMQ application. In contrast, C57BL/6 J mice received topical doses of 20 mg/cm^2^ of 5% IMQ cream (or vehicle) on the back for 6 consecutive days.

### Scratching assessment

Twenty-four hours after the last IMQ application (Day 10)—rats (or mice) were habituated to the recording chamber for 60 min before animal behaviours were captured by videotape. Scratching was recorded on day ten. Behaviours were captured alone, undisturbed in the observation room for 120 min. A bout of scratching was defined as one or more rapid movements of the hind-paws directed toward and contacting the IMQ/Vaseline^®^ treated area—ending with licking or biting of toes and/or placement of the hind-paw on the floor. Hind-paw movements directed away from the injection site (e.g., ear scratching) and grooming movements were not counted. The degree of scratching was quantified as the total number of bouts of scratching in the observation period.

### Psoriasis-like inflammation assessment

Severity of the inflammation of the back skin was assessed by a modified Psoriasis Area and Severity Index (PASI) score. Erythema, scaling and thickening were assigned values of 0 to 4 as follows: 0-none; 1-slight; 2-moderate; 3-marked; 4-very marked. Scoring was prior to the daily IMQ topical applications on Day 1, 4, 6, 8 and 10. The cumulative score served to indicate the severity of inflammation.

### Endpoint blood sampling and skin sampling

Approximately 1 mL of whole blood was taken by sublingual vein puncture in anaesthetized rats, transferred into serum gel tubes containing clot activator and inverted to allow mixture for 30 min, room temperature. Samples were then centrifuged for 10 min at 4 °C with resulting serum transferred to polypropylene tubes and stored at − 80 °C for cytokine analysis. Back skin of about 2 × 2 cm dissected from the subcutaneous tissue, were rinsed in physiological saline and transferred to 4% methanol-free paraformaldehyde in 0.1 M PBS pH 7.4 for a 48-h fixation, rinsed again in PBS and stored at 4 °C for immunohistochemistry.

### Immunohistochemistry

Fixed back skin samples from Wistar rats were embedded, sectioned, stained and imaged using MultiBrain^®^ Technology (Neuroscience Associates, Knoxville, TN), during which multiple skin samples are embedded within gelatin blocks providing uniform exposure to sectioning and staining conditions. Cryo-sectioning was performed at 40 µm in the cross-sectional planes through the embedded skin. Sections were stained with hematoxylin and eosin and mounted on gelatinized glass slides. Each slide was digitally imaged at 20 ×magnification.

### Cytokine analysis

Serum analytes were analyzed for IL-6, IL-1β, IL-17A and TNF-α using a combination of V-Plex and U-Plex Meso Scale Discovery electro chemiluminescent (MSD-ECL) multiplex panels (Sword Bio, Chicago, IL, USA).

### Data analysis

Statistical significance was determined using the Student’s *t* test (GraphPad Prism Software 9, La Jolla, CA, USA) followed by post hoc Mann–Whitney test. The differences between data groups were considered significant if adjusted *P* < 0.05. An outlier test was applied before statistical analysis of each set of data using the Robust Regression and Outlier Removal (ROUT) method (Q = 1%) (GraphPad Prism Software 9, La Jolla, CA, USA) [7].

## Results

**Fig. 2:**
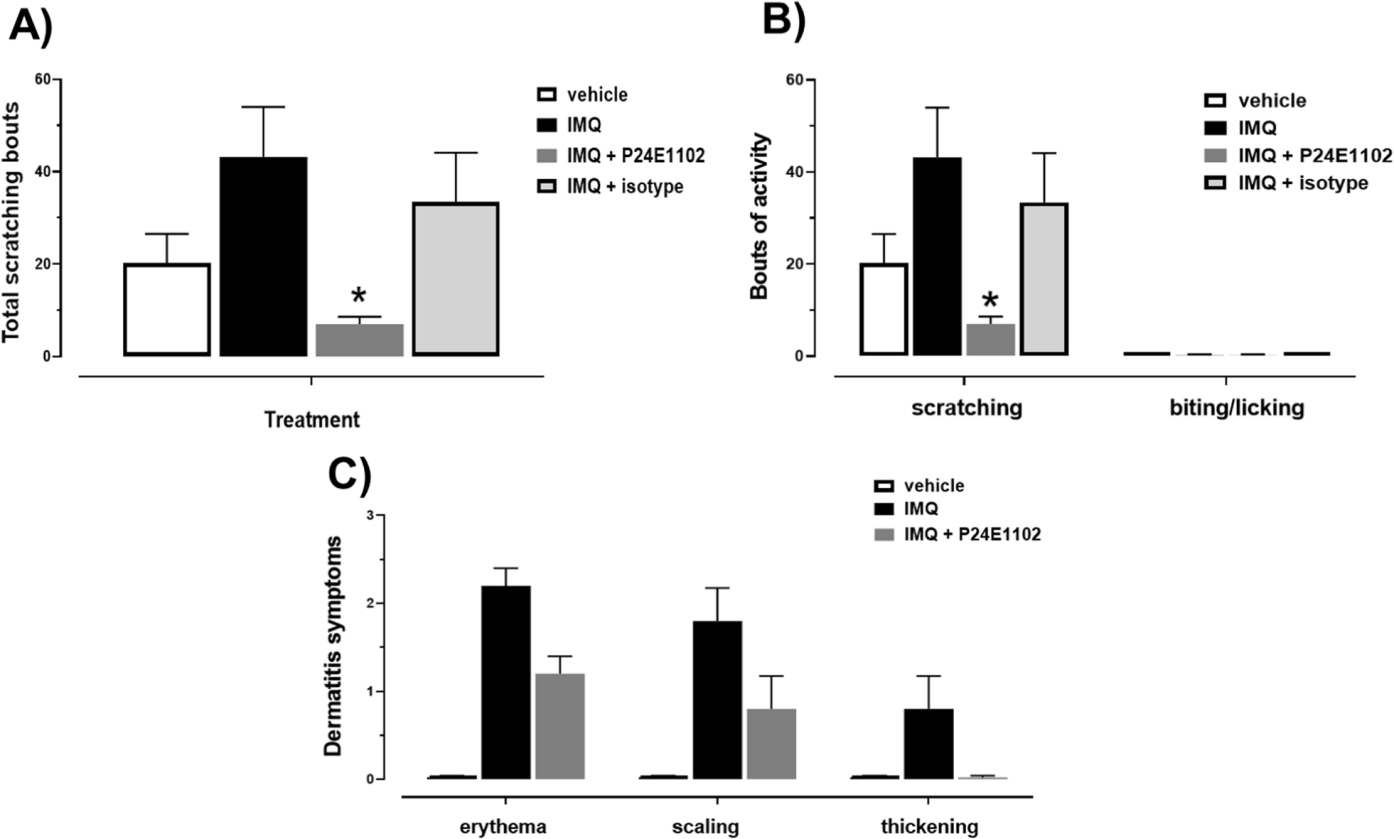
Effect of IMQ administration on total scratching bouts and severity of psoriasis-like activity in F2RL1 rats. **A** Rats with total scratching bouts treated with IMQ were significantly higher than animals exposed to vehicle (n = 5/group). Rats with scratching bouts after exposure to IMQ show decreased scratching upon administration of P24E1102 at 30 mg/kg IV at Day 10 as compared with vehicle and isotype control-dosed rats (P < 0.05) (n = 5/group). **B** IMQ induces scratching activity, but not biting or licking. Scratching is the main activity observed. **C** Rats with dermatology severity symptoms of erythema, scaling and thickening of lesions after exposure to IMQ show decreased severity upon administration with P24E1102. Data is expressed as the mean (SEM). Asterisk (*) denotes P values of IMQ compared to vehicle, *P < 0.05 (Student’s *t*-test, followed by Mann–Whitney post-test

**Fig. 3:**
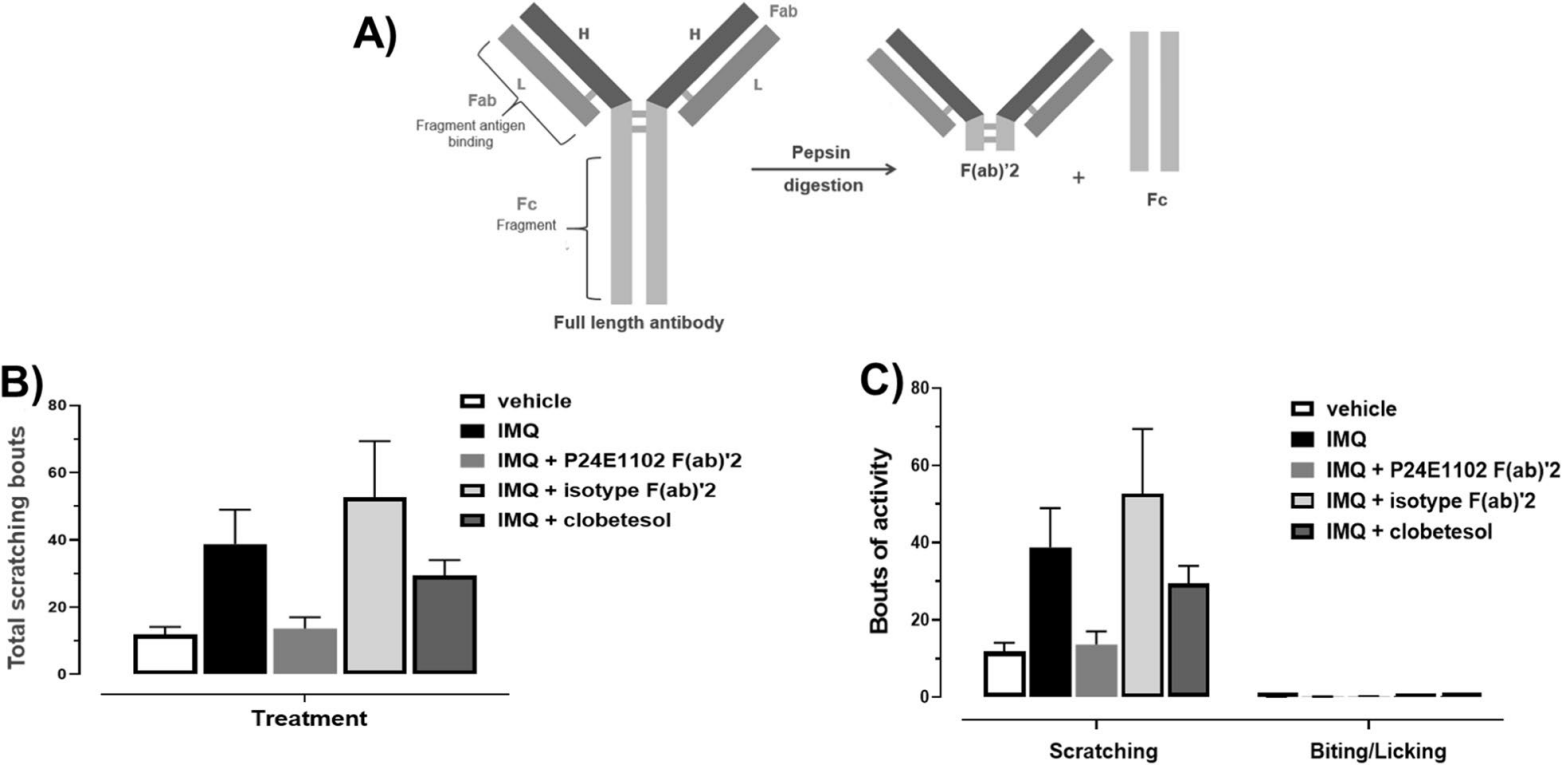
Confirming specific targeting effects of P24E1102 **A** Rats administered with the negative control isotype antibody may show decreased severity scores after exposure to IMQ and may induce activation of non-antigen specific Fc receptors interactions. Therefore, we made F(ab)’2 antibody fragments **A** and retested them in the IMQ rat model. **B** Target specific attenuation of IMQ-induced scratching activity with P24E1102 F(ab)’2 30 mg/kg IV similar to the whole IgG molecule, while the isotype F(ab)’2 showed no targeting effect (n = 5/group). **C** IMQ induces scratching activity, but not biting or licking. Data is expressed as the mean (SEM). Statistical significance was determined using Student’s *t* test followed by Mann–Whitney post-test

Using an established mouse protocol (six consecutive daily topical IMQ doses) [19–22] we found PASI scores increased steadily over the seven-day period and scratching increased significantly (> 3.5-fold, P =  < 0.0001) with typically seen lesions [Additional files 1, 2). Our rat paradigm uses daily topically applied IMQ for a longer period than most mouse models (9 consecutive days: 37.5 mg/cm^2^ of skin). (Fig. 1A) Development of psoriasis-like dermatitis appeared Day 4 through Day 10. Behavioral scratching evaluation was evaluated on Day 10 (Fig. 1B). Scratching bouts achieved were measurable and consistent as shown in the compiled control values of scratching with vehicle and diseased (IMQ-induced) treatments. The number of scratching bouts was significantly (P < 0.0001) higher in the IMQ-treated vehicle animals in both Wistar and F2RL1 knockin animals by threefold (Fig. 1C, D). We also observed a similar induction in male rats. Next, we evaluated P24E1102, isotype control or vehicle dosed intravenous Day 1, 3, 5 in F2RL1 knockin rats. P24E1102 significantly attenuated bouts of scratching (Fig. 2A). The total bouts of scratching activity can be further classified into scratching or biting/licking behaviours [Fig. 2B]. P24E1102 appears to impact dermatitis symptoms of erythema, scaling and thickening of skin. (Fig. 2C), In addition to demonstrating the efficacy of antagonizing PAR2 activity by P24E1102, we modified the antibody by enzymatically removing the Fc region to create F(ab)’2 (Fig. 3A) which would eliminate any possible Fc interactions/activity that might be contributing to its efficacy. Administration of the F(ab)’2 was given Day 1, 3, 5, 7 and 9 prior to IMQ application. As shown in (Fig. 3B), P24E1102 Fab’2 retained its attenuation of IMQ-induced total scratching bouts demonstrating the attenuation was due to the antagonistic PAR2 moiety (Fig. 3C). This supports the specificity of P24E1102 antagonism, whilst removing the non-specific immune interactions often associated with the Fc moiety.

Earlier studies collectively have reported several cytokines elevated in rodents following IMQ-induction also commonly seen in human psoriasis patients [8–11]. Therefore, we measured the detectable inflammatory cytokine levels of IL-1β, IL-6, IL-17A and TFNα (Table 1) in the serum of both IMQ-treated and vehicle-treated rats. We identified significant increases above non-treated with TFNα in the IMQ-treated animals, comparable to what people have found in mouse serum. (Additional file 3) Hallmarks of psoriasiform dermatitis morphological skin reactions were observed in histology by epidermal thickness in all IMQ treated condition and subsequent reduction in thickening with P24E1102 (Additional file 4).

**Table 1:** Comparison of published induced inflammatory cytokines interleukin IL-6, IL-1β, IL-17A and TNF-α performed on both skin and serum samples from rat, mouse and human using a combination of ELISA methods and MSD-ECL assay methods

Cytokine	IMQ rat induced serum	IMQ mouse induced serum	Psoriasis patient serum	IMQ rat induced skin	IMQ rat induced skin
IL-1β	−	−	−	−	+
IL-6	−	−	+	−	+
IL-17A	−	−	+	−	+
TNFα	+	+	+	+	+
Detection	MSD-ECL	ELISA	ELISA/MSD-ECL	ELISA	ELISA
Tissue	Serum	Serum	Serum	Back skin	Ear skin
References	^a^	[9]	[8]	[10]	[11]

*NT* not tested

− negative or not tested

+ singnificant increases observed above not tested

^a^Our results

## Discussion

In this study, we documented the effects of IMQ on rat skin and characterized the use of this treatment to establish a model resembling human psoriasis. Furthermore, we characterized the effect of IMQ on pruritus by documenting scratching and used this behaviour to investigate the effectiveness of topical corticosteroid treatment and an investigational P24E1102 antibody on preventing/reducing skin lesions and scratching.

Interestingly, P24E1102 selectively attenuated only scratching, while biting/licking (surrogate measure of behavioural nociceptive pain) are not present with this IMQ induction. The ability of P24E1102 to attenuate only scratching and not the PASI score seems to indicate it affects largely a neuronal based mechanism as opposed immune mediated itch [12].

The well documented topical application of IMQ elicits an inflammatory reaction of erythema, scaling, and epidermal thickening in murine skin. These features have the appeal of being a fast and reproducible to model. While histological features and PASI scores are useful to validate the IMQ model as surrogate markers of skin inflammation, these measures can easily be misinterpreted or over relied on [13]. Similarly, the modified clinical PASI is [1] susceptible to an observer bias that cannot be ignored. While itch sensation cannot be accessed by the observer, scratching behaviour can be observed, recorded, and quantified. This reliability is supported by other researchers due to its usefulness in the clinical setting [14]. Therefore, scratching is a useful, unbiased method to assess the effectiveness of potential antipruritic therapies. Since we observed efficacy in reducing scratching with the P24E1102 antibody is consistent with the mechanisms of itch in this model involving PAR2 signalling. This itch signal could underlie the non-TLR-independent mechanisms in IMQ induction implied by several studies [15, 16], Patent Application US20200246305A1.

## Limitations

Despite the novelty of using a genetic model rat here to provide insight into the proposed pathways of itch mechanisms, there are some potential limitations to our studies. While we supported the involvement a PAR2 mechanism in IMQ mediated itch, further studies are required to test direct involvement of specific reported partners like TRPV3 and PAR2 [16–18] following cytokine induction. Second, we are aware of excipients such as isostearic acid [15] that can have contributing effects to IMQ as well. Considerations to further isolate its effects in our model can improve pathway understanding in this model. [23–27] Finally, additional investigations substantiating scratching in other in vivo models of itch further highlight its behavioural importance endpoint to the evaluation in skin disease [14].

### Supplementary Information

**Additional file 1: Fig. S1.** Mouse study design.

**Additional file 2: Fig. S2.** Images of psoriatic lesions of dorsal portion on application of imiquimod.

**Additional file 3: Fig. S3.** Quantification of serum analytes produced in Wistar rat after 9 days of IMQ induction as measured by MSD-ECL technology.

**Additional file 4: Fig. S4.** Histopathological view of psoriatic skin lesions of dorsal portion on imiquimod application.

## Data Availability

The datasets used and/or analyzed during the current study are available from the corresponding author on reasonable request.

## Abbreviations

IMQ: Imiquimod
PBS: Phosphate buffered saline
PASI: Psoriasis area and severity index

## References

[1] van der Fits L, Mourits S, Voerman JS, Kant M, Boon L, Laman JD (2009). Imiquimod-induced psoriasis-like skin inflammation in mice is mediated via the IL-23/IL-17 axis. J Immunol.

[2] Swindell WR, Johnston A, Carbajal S, Han G, Wohn C, Lu J (2011). Genome-wide expression profiling of five mouse models identifies similarities and differences with human psoriasis. PLoS ONE.

[3] Gudjonsson JE, Johnston A, Dyson M, Valdimarsson H, Elder JT (2007). Mouse models of psoriasis. J Invest Dermatol.

[4] Yang W, Zhou B, Liu Q, Liu T, Wang H, Zhang P (2022). A murine point mutation of sgpl1 skin is enriched with Vgamma6 IL17-producing cell and revealed with hyperpigmentation after imiquimod treatment. Front Immunol.

[5] Tivoli YA, Rubenstein RM (2009). Pruritus: an updated look at an old problem. J Clin Aesthet Dermatol.

[6] Smajlovic A, Haveric A, Alic A, Hadzic M, Mujezinovic I, Lojo-Kadric N (2021). Molecular and histopathological profiling of imiquimod induced dermatosis in Swiss Wistar rats: contribution to the rat model for novel anti-psoriasis treatments. Mol Biol Rep.

[7] Motulsky HJ, Brown RE (2006). Detecting outliers when fitting data with nonlinear regression—a new method based on robust nonlinear regression and the false discovery rate. BMC Bioinform.

[8] Bai F, Zheng W, Dong Y, Wang J, Garstka MA, Li R (2018). Serum levels of adipokines and cytokines in psoriasis patients: a systematic review and meta-analysis. Oncotarget.

[9] Jabeen M, Boisgard AS, Danoy A, El Kholti N, Salvi JP, Boulieu R (2020). Advanced characterization of imiquimod-induced psoriasis-like mouse model. Pharmaceutics.

[10] Imbertson LM, Beaurline JM, Couture AM, Gibson SJ, Smith RM, Miller RL (1998). Cytokine induction in hairless mouse and rat skin after topical application of the immune response modifiers imiquimod and S-28463. J Invest Dermatol.

[11] Parmar KM, Jagtap CS, Katare NT, Dhobi M, Prasad SK (2021). Development of a psoriatic-like skin inflammation rat model using imiquimod as an inducing agent. Indian J Pharmacol.

[12] Schmelz M (2021). How do neurons signal itch?. Front Med.

[13] Hawkes JE, Gudjonsson JE, Ward NL (2017). The snowballing literature on imiquimod-induced skin inflammation in mice: a critical appraisal. J Invest Dermatol.

[14] Smith MP, Ly K, Thibodeaux Q, Weerasinghe T, Wu JJ, Yosipovitch G (2019). Emerging methods to objectively assess pruritus in atopic dermatitis. Dermatol Ther.

[15] Walter A, Schafer M, Cecconi V, Matter C, Urosevic-Maiwald M, Belloni B (2013). Aldara activates TLR7-independent immune defence. Nat Commun.

[16] Zhao J, Munanairi A, Liu XY, Zhang J, Hu L, Hu M (2020). PAR2 mediates Itch via TRPV3 signaling in keratinocytes. J Invest Dermatol.

[17] Huang SM, Lee H, Chung MK, Park U, Yu YY, Bradshaw HB (2008). Overexpressed transient receptor potential vanilloid 3 ion channels in skin keratinocytes modulate pain sensitivity via prostaglandin E2. J Neurosci.

[18] Wimalasena NK, Milner G, Silva R, Vuong C, Zhang Z, Bautista DM (2021). Dissecting the precise nature of itch-evoked scratching. Neuron.

[19] Turner PV, Pang DS, Lofgren JL (2019). A review of pain assessment methods in laboratory rodents. Comp Med.

[20] Swindell WR, Michaels KA, Sutter AJ, Diaconu D, Fritz Y, Xing X (2017). Imiquimod has strain-dependent effects in mice and does not uniquely model human psoriasis. Genome Med.

[21] Schon MP, Manzke V, Erpenbeck L (2021). Animal models of psoriasis-highlights and drawbacks. J Allergy Clin Immunol.

[22] Sakai K, Sanders KM, Youssef MR, Yanushefski KM, Jensen L, Yosipovitch G (2016). Mouse model of imiquimod-induced psoriatic itch. Pain.

[23] Okasha EF, Bayomy NA, Abdelaziz EZ (2018). Effect of topical application of black seed oil on imiquimod-induced psoriasis-like lesions in the thin skin of adult male albino rats. Anat Rec.

[24] Langford DJ, Bailey AL, Chanda ML, Clarke SE, Drummond TE, Echols S (2010). Coding of facial expressions of pain in the laboratory mouse. Nat Methods.

[25] Kisipan ML, Ojoo RO, Kanui TI, Abelson KSP (2020). Imiquimod does not elicit inflammatory responses in the skin of the naked mole rat (Heterocephalus glaber). BMC Res Notes.

[26] Horvath S, Komlodi R, Perkecz A, Pinter E, Gyulai R, Kemeny A (2019). Methodological refinement of Aldara-induced psoriasiform dermatitis model in mice. Sci Rep.

[27] Deuis JR, Dvorakova LS, Vetter I (2017). methods used to evaluate pain behaviors in rodents. Front Mol Neurosci.

