# Supplementary material for: Imiquimod-induced pruritus in female wild-type and knockin Wistar rats: underscoring behavioral scratching in a rat model for antipruritic treatments
Source: BMC Res Notes. 2023 Nov 25;16:348. doi: 10.1186/s13104-023-06627-1 (PMC10675923; doi:10.1186/s13104-023-06627-1)
Supplement: Supplementary file 1 — Additional file 1: Fig. S1. Mouse study design. [file 13104_2023_6627_MOESM1_ESM.pdf]

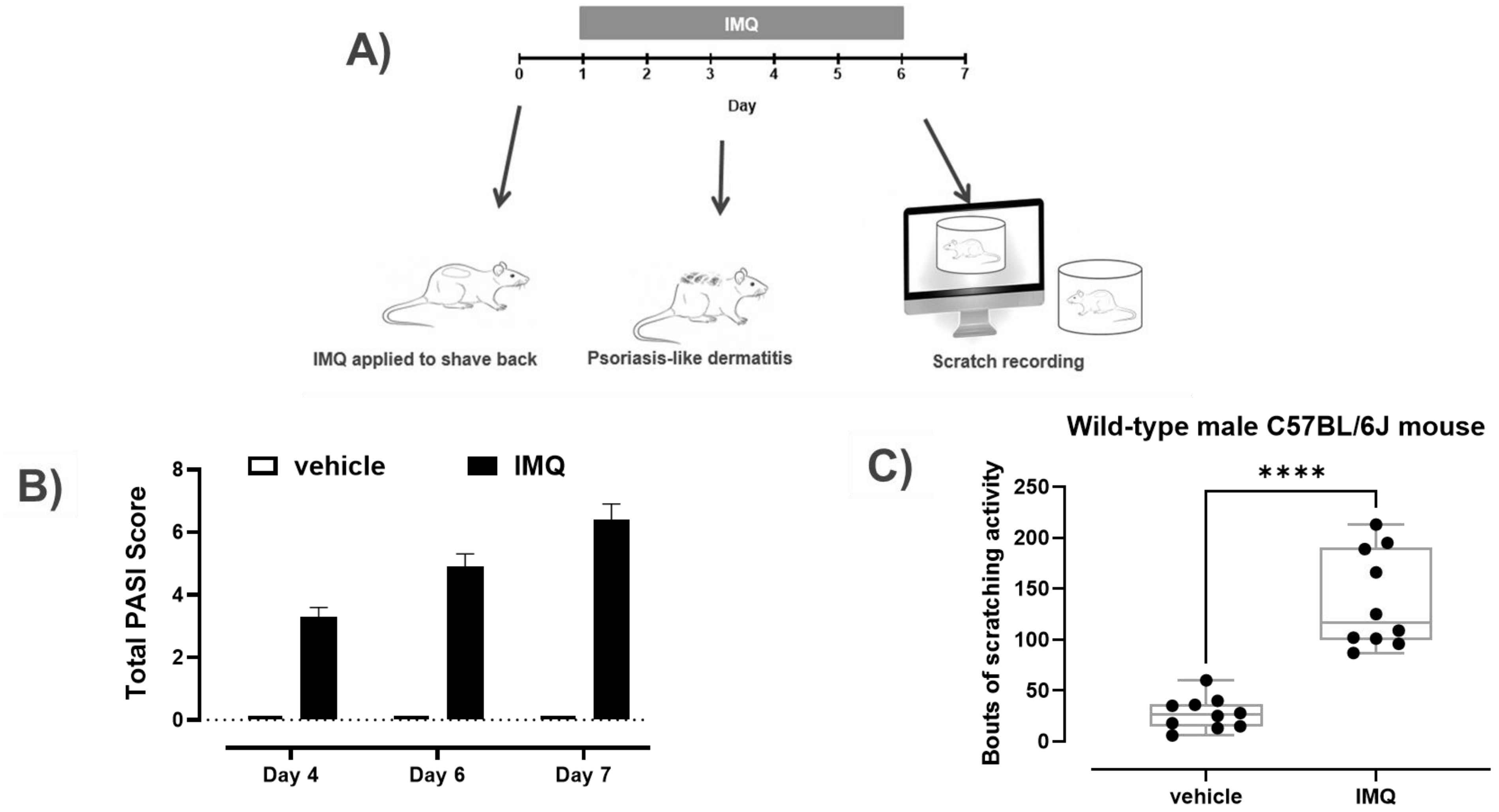

Fig. S1 Mouse study design. A) C57BL/6J mice received from seven daily topical administrations of imiquimod (IMQ) or vehicle (Vaseline®). Test antibodies or vehicle formulations were administered on Day 1, 3, and Day 6. Development of psoriasis-like dermatitis B) appeared starting Day 4 continuing through Day 7. On Day 7, severity of the back inflammation was monitored on a modified clinical Psoriasis Area and Severity Index (PASI) scoring system. Scratching behavior was captured by videotape and serum and back skin samples were collected for cytokine measurements and immunohistochemistry to visualize IMQ induced morphological changes. C) Number of scratching bouts was significantly (P<0.0001) higher than animals exposed to vehicle in wild-type male mice from various studies under control conditions (n = 10 total). Data is expressed as the mean (SEM). Statistical significance was determined using Student's *t* test followed by Mann-Whitney post-test.
