# Supplementary material for: Imiquimod-induced pruritus in female wild-type and knockin Wistar rats: underscoring behavioral scratching in a rat model for antipruritic treatments
Source: BMC Res Notes. 2023 Nov 25;16:348. doi: 10.1186/s13104-023-06627-1 (PMC10675923; doi:10.1186/s13104-023-06627-1)
Supplement: Supplementary file 2 — Additional file 2: Fig. S2. Images of psoriatic lesions of dorsal portion on application of imiquimod. [file 13104_2023_6627_MOESM2_ESM.pdf]

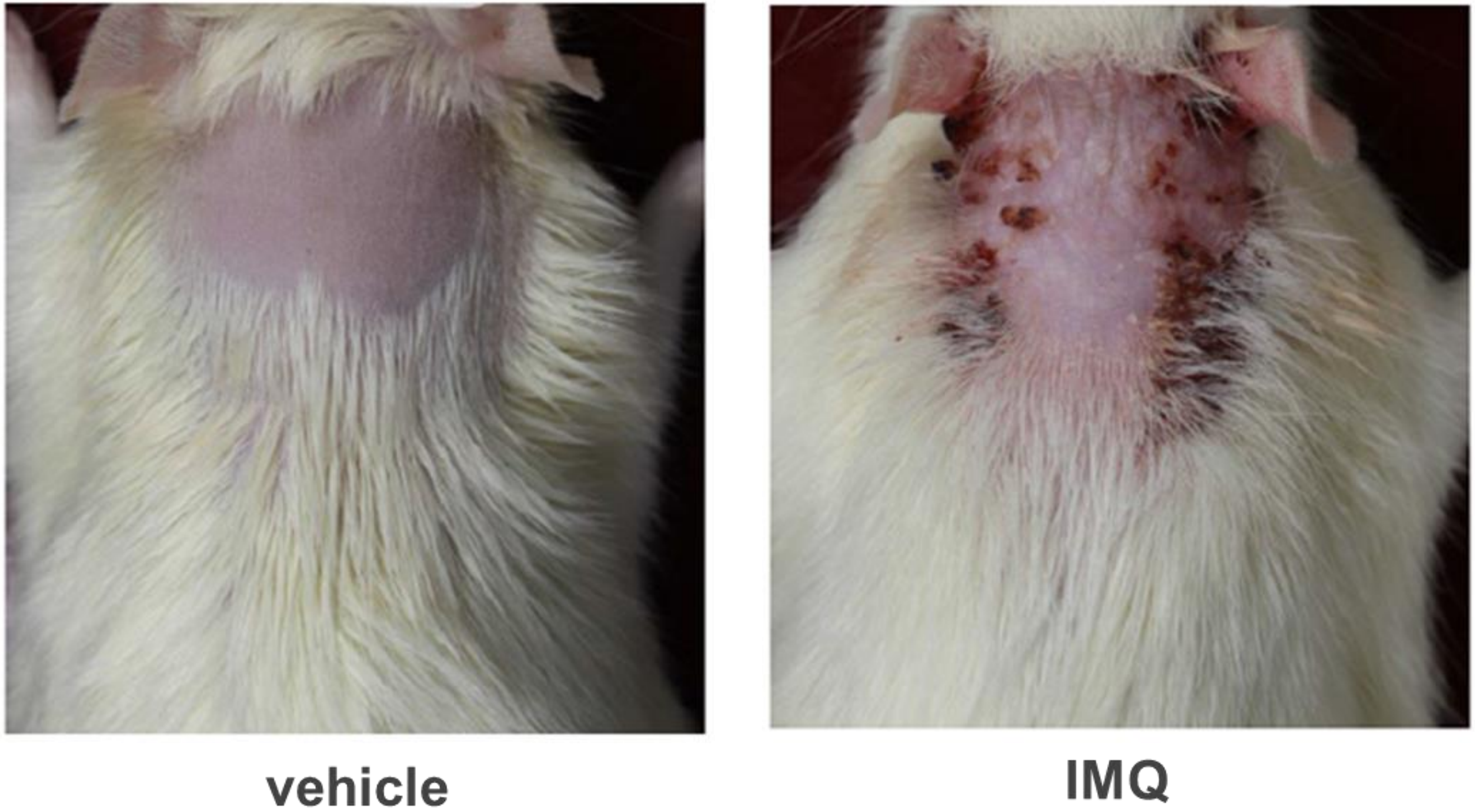

Fig. S2 Images of psoriatic lesions of dorsal portion on application of imiquimod. Images of mouse 7 days after daily vehicle control (left) and imiquimod 20 mg/cm^2 (right). The IMQ-treated mouse demonstrates the typical lesions observed.
