# Supplementary material for: Imiquimod-induced pruritus in female wild-type and knockin Wistar rats: underscoring behavioral scratching in a rat model for antipruritic treatments
Source: BMC Res Notes. 2023 Nov 25;16:348. doi: 10.1186/s13104-023-06627-1 (PMC10675923; doi:10.1186/s13104-023-06627-1)
Supplement: Supplementary file 3 — Additional file 3: Fig. S3. Quantification of serum analytes produced in Wistar rat after 9 days of IMQ induction as measured by MSD-ECL technology. [file 13104_2023_6627_MOESM3_ESM.pdf]

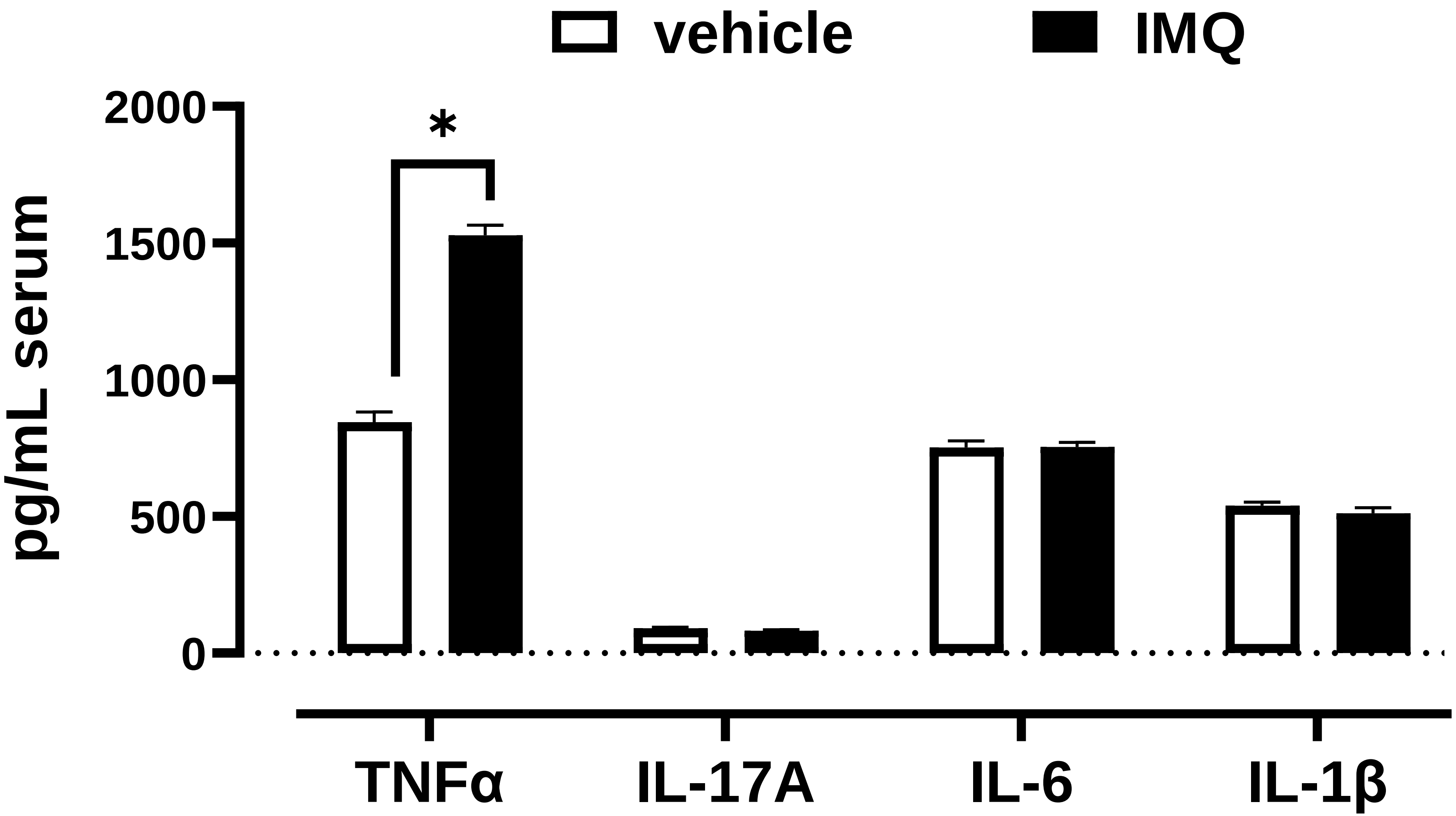

Fig. S3 Quantification of serum analytes produced in Wistar rat after 9 days of IMQ induction as measured by MSD-ECL technology. Data is expressed as the mean (SEM). Statistical significance was determined using Student's *t* test followed by Mann-Whitney post-test. Data is expressed as the mean (SEM). Asterisk (*) denotes P values of IMQ compared to vehicle, $^{*}P < 0.05$.
