# Supplementary material for: Imiquimod-induced pruritus in female wild-type and knockin Wistar rats: underscoring behavioral scratching in a rat model for antipruritic treatments
Source: BMC Res Notes. 2023 Nov 25;16:348. doi: 10.1186/s13104-023-06627-1 (PMC10675923; doi:10.1186/s13104-023-06627-1)
Supplement: Supplementary file 4 — Additional file 4: Fig. S4. Histopathological view of psoriatic skin lesions of dorsal portion on imiquimod application. [file 13104_2023_6627_MOESM4_ESM.pdf]

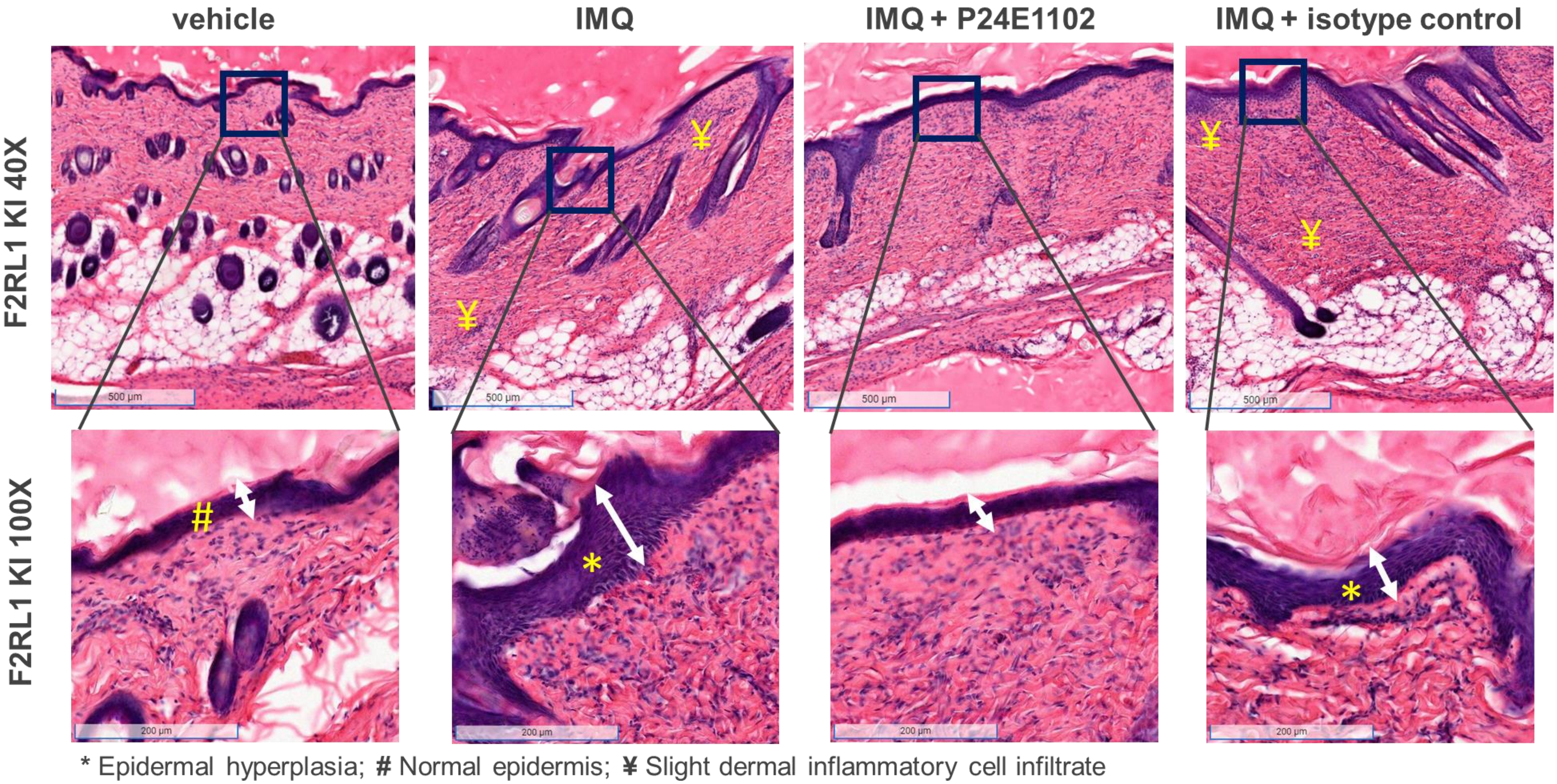

Fig. S4 Histopathological view of psoriatic skin lesions of dorsal portion on imiquimod application. In figure above (Left to right; top panel 40X magnification, bottom panel 100X magnification) vehicle, IMQ, IMQ+ P24E1102 at 30 mg/kg, IMQ with PE24E1102 treatment appears to attenuate thickening compared to IMQ treatment alone.
